# The laboratory rat: Age and body weight matter

**DOI:** 10.17179/excli2021-4072

**Published:** 2021-09-23

**Authors:** Asghar Ghasemi, Sajad Jeddi, Khosrow Kashfi

**Affiliations:** 1 Endocrine Physiology Research Center, Research Institute for Endocrine Sciences, Shahid Beheshti University of Medical Sciences, Tehran, Iran; 2 Department of Molecular, Cellular and Biomedical Sciences, Sophie Davis School of Biomedical Education, City University of New York School of Medicine, New York, USA

**Keywords:** age, animal experimentation, body weight, developmental stage, humans, laboratory animals, rat

## Abstract

Animal experimentation helps us to understand human biology. Rodents and, in particular, rats are among the most common animals used in animal experiments. Reporting data on animal age, animal body weight, and animal postnatal developmental stages is not consistent, which can cause the failure to translate animal data to humans. This review summarizes age-related postnatal developmental stages in rats by addressing age-related changes in their body weights. The age and body weight of animals can affect drug metabolism, gene expression, metabolic parameters, and other dependent variables measured in animal studies. In addition, considering the age and the body weight of the animals is of particular importance in animal modeling of human diseases. Appropriate reporting of age, body weight, and the developmental stage of animals used in studies can improve animal to human translation.

## Introduction

Using animals to model human anatomy and physiology dates back to 600 BC (Ericsson et al., 2013[25]). Animal experiments help us to understand human biology (Bahadoran et al., 2020[7]) and are important for understanding the pathophysiological and therapeutic basis of human diseases (Flórez-Vargas et al., 2016[28]). The importance of animal research in biomedical sciences is evident when we know that about 90 % of Nobel Prizes in Physiology or Medicine have been related to research done on animals (Pasquali, 2018[79]). It has been estimated that more than 115 million animals were used for research purposes in 2005 (Taylor et al., 2008[111]), and the number is increasing (Goodman et al., 2015[33]; Hudson-Shore, 2016[46]). Rodents are the most common animals used in animal experimentations (Kilkenny et al., 2009[51]; Hatton et al., 2015[36]; Jackson et al., 2017[49]), constituting about 80 % of experimental animals (Sengupta, 2013[99]). Among laboratory animals, rats are extensively used in different medical disciplines (Clause, 1993[18]; Gille et al., 1994-1996[32]), particularly in toxicology (Beckman and Feuston, 2003[9]; Vidal, 2017[115]), obesity (Reed et al., 2011[92]), social stress experiments (Buwalda et al., 2011[16]), osteoporosis (Yousefzadeh et al., 2020[119]), diabetes (Lane, 1997[57]), and neurobiology (Romijn et al., 1991[95]). Of 51 known species (Andreollo et al., 2012[6]) and more than 1000 known rat strains (Reed et al., 2011[92]), Wistar and Sprague-Dawley rats are mostly used in animal studies (Andreollo et al., 2012[6]).

Some evidence strongly suggests that animal studies are mostly not translated to humans (Pound et al., 2004[87]; Akhtar, 2015[2]), with more than 80 % of the reported safe and effective treatments in animal studies failing to translate to humans (Perrin, 2014[81]). In addition to the intrinsic limitations of animal models, poor design and reporting of animal studies are major causes of poor concordance between preclinical and clinical outcomes (Perrin, 2014[81]; Bahadoran et al., 2020[7]). This has led to a reproducibility crisis in biomedical research (Osborne et al., 2018[76]), particularly in preclinical research using animal models (Collins and Tabak, 2014[19]). The provision of basic variables including age and body weight of animal used is the starting point of enabling replication (NRC, 2011[72]). In addition, considering the difference between rodents and human timescales helps translate experimental treatment into clinical practice (Agoston, 2017[1]).

Body weight, age, and developmental stage of the animals used are characteristics that can affect the study's results (Kilkenny et al., 2009[51]; Jackson et al., 2017[49]). The age and the body weight of the animals can affect drug metabolism, gene expression, metabolic parameters, and other dependent variables measured in animal studies (McCutcheon and Marinelli, 2009[65]; Ihedioha et al., 2013[47]; Jackson et al., 2017[49]). The animal's age plays an important role in modeling human diseases; examples across different fields are presented here. If the study duration in rodents takes ≥ 3 months, results can be affected by reproductive changes (Vidal, 2017[115]). In addition, rats aged 6-9 months are most suitable for studying osteoporosis because of a stable level of bone turnover; however, rats aged under 6 months or over 9 months are not ideal because of the high bone growth rate and ageing process respectively (Yousefzadeh et al., 2020[119]). In rat models of diabetes, β-cell mass increases almost linearly from late fetal to postnatal day (PND) 100 in normal Sprague-Dawley rats (Finegood et al., 1995[26]), and sensitivity to the diabetogenic effects of streptozotocin is inversely related to age in the Wistar rats (Masiello et al., 1979[63]). Age-related differences in response to alcohol have also been reported; compared to adults, aged rats and humans are more sensitive to alcohol-induced motor and cognitive impairments (Squeglia et al., 2014[104]). Overlooking the age of the animals in design and reporting of animal studies is also a significant factor in failure in translation from animals to humans in neurological disorders (Sun et al., 2020[108]).

According to guidelines for reporting animal studies, animals' body weight and age need to be reported (Hooijmans et al., 2010[42]; Osborne et al., 2018[76]; Percie du Sert et al., 2020[80]; Nagendrababu et al., 2021[69]). However, despite being available, these data frequently are not reported (Kilkenny et al., 2009[51]; Jackson et al., 2017[49]), which can cause failure to translate animal data to the human (Ioannidis, 2012[48]). Of 271 papers reporting experimental results on mice, rats, and non-human primates, age and body weights of the animals had not been reported in 57 %, and 54 % of papers, respectively, and 24 % of papers reported neither the age nor the body weight (Kilkenny et al., 2009[51]); of 113 studies conducted in rats, 67.3 % did not report age, and 16.8 % did not report the body weights (Kilkenny et al., 2009[51]). Results of a text mining of over 15311 articles that used mice indicate that 38.6 % of the papers did not note the age of the animals used. This bias varied across biomedical fields, with missing information about the age being 20.9 % in diabetes mellitus, 29.7 % in neurological disorders, 30.23 % in cardiovascular diseases, 33.7 % in infectious diseases, 35.0 % in lung diseases, and 37.2 % in cancer studies (Flórez-Vargas et al., 2016[28]). This reporting bias, i.e., the absence of essential results from the study (O'Connor and Sargeant, 2014[74]), is mainly due to the fact that the potential values and effects of these ancillary variables on study outcomes are not recognized (Gaines Das, 2002[29]; Flórez-Vargas et al., 2016[28]). This paper reviews age-related postnatal developmental stages in rats addressing age-related changes in their body weights to provide a basis for better design and report of animal studies and help explain acquired data. 

## Age-Related Postnatal Development in Rats

Rats are born after 21-22 days of gestation period (Romijn et al., 1991[95]; Picut and Ziejewski, 2018[86]). Rat is an altricial species (Henning, 1981[38]), i.e., they are delivered in a very immature condition. It has been suggested that at PND 12-13, the rat neocortex is developmentally comparable with a newborn human (Romijn et al., 1991[95]). Therefore, it is said that rats are born at PND 7 (Nuñez et al., 2003[73]) or PND 12 (Quinn, 2005[89]). In fact, PND 1-10 in rats is comparable with 23-40 gestational weeks in humans (Semple et al., 2013[98]). Postnatal development in rats can be considered from different points of view, including sexual maturation and nutritional behavior.

### Sexual development

Sexual development in rats has five stages (Table 1(Tab. 1); References in Table 1: Beckman and Feuston, 2003[9]; Bell, 2018[10]; Bjorklund, 2015[13]; Marty et al., 2003[62]; Picut and Ziejewski, 2018[86]; Quinn, 2005[89]; Semple et al., 2013[98]; Stanley and Shetty, 2004[105]; Vidal, 2017[115]): (1) neonatal period, (2) infantile period, (3) juvenile period, (4) peripubertal period (Picut and Ziejewski, 2018[86]), and (5) adolescence period (Bell, 2018[10]). 

The neonatal period is an extension of gestation (Bell, 2018[10]). In males, it is characterized by small seminiferous tubules, regression of Leydig cells, and mitosis in Sertoli and spermatogonial cells; in addition, there are no spermatocytes or spermatids (Picut and Ziejewski, 2018[86]). In female rats, apoptosis of oogonia and primordial follicles are predominant features of the neonatal period (Picut et al., 2015[83]).

The infantile period is a time of advancing sensory development (Bell, 2018[10]) and is characterized by proliferation of Sertoli, Leydig, and spermatogonial cells and not spermatocyte formation; at the end of this period, blood-testis barrier is formed (Picut and Ziejewski, 2018[86]). In female rats, maturation of secondary and early antral follicles and the appearance of zona pellucida are salient features during the infantile period (Picut et al., 2015[83]). 

The juvenile period is characterized by spermatogenesis and the beginning of spermiogenesis in male rats (Picut and Ziejewski, 2018[86]), apoptosis of granulosa cells and the enlargement of antral follicles in female rats (Picut et al., 2015[83]). 

The peripubertal period occurs from the onset of puberty, where circulating gonadal hormones start to rise, leading to sexual/reproductive maturation (Bell, 2018[10]; Picut and Ziejewski, 2018[86]). Puberty is the developmental stage in which sexual development is completed, and reproductive capacity or fertility is achieved (Ojeda et al., 1980[75]; Vidal, 2017[115]; Bell, 2018[10]). Terminology related to puberty is inconsistently used (Laffan et al., 2018[56]); in some publications, the term puberty is used synonymously with peripubertal (Laffan et al., 2018[56]; Picut and Ziejewski, 2018[86]) to stress the transitional nature of the pubertal period (Laffan et al., 2018[56]). Sometimes, the last few days of the peripubertal period are considered as puberty (Picut and Ziejewski, 2018[86]). Collectively, puberty is a period of transition to sexual maturity (Laffan et al., 2018[56]) or transition from childhood to adulthood (De Silva and Tschirhart, 2016[22]).

The onset of puberty in male rats is when mature spermatozoa are first seen in seminiferous tubules (Picut and Ziejewski, 2018[86]). Sometimes, puberty in male rats is defined as the shorter time of preputial separation (PPS), i.e., separation of the foreskin of the penis from the glans penis (Picut and Ziejewski, 2018[86]); PPS is an index of the onset of puberty in male rats (Korenbrot et al., 1977[52]) and is the best external indication of the pubertal period in male rats (Picut and Ziejewski, 2018[86]) (Table 2(Tab. 2); References in Table 2: Campion et al., 2013[17]; Gaytan et al., 1988[30]; Korenbrot et al., 1977[52]; Laffan et al., 2018[56]; Lewis et al., 2002[60]; Rivest, 1991[93]; Tinwell et al., 2002[112]; Vidal, 2017[115]). PPS in humans begins during late gestation and completes from 9 months to 3 years of age (Marty et al., 2003[62]). In female rats, the vaginal opening is an observable sign for the onset of puberty (Vidal, 2017[115]; Laffan et al., 2018[56]), followed by irregular estrous cycles for weeks before full sexual maturity (Laffan et al., 2018[56]). Time of vaginal opening is species-dependent, and there is also individual difference (Rivest, 1991[93]; Lewis et al., 2002[60]) (Table 2(Tab. 2)). Vaginal opening in humans occurs in the prenatal period (Laffan et al., 2018[56]).

The end of puberty (sexual maturation) in male rats is when all seminiferous tubules have complete spermiogenesis, and mature spermatozoa are readily visible in the epididymis (Picut and Ziejewski, 2018[86]) or vas deferens (Bell, 2018[10]). In female rats, 4- to 5-day regular estrous cycles mark the completion of puberty (Bell, 2018[10]). 

The period between the onset of sexual maturity and attainment of adult roles is called late adolescence, which is a time of increased reward-seeking and social reorientation; these behaviors are mediated by the maturation of affective brain areas (McCutcheon and Marinelli, 2009[65]; Bell, 2018[10]). 

It should be noted that there are species and individual differences between timepoints presented for sexual development; this is partly due to species differences (Table 2(Tab. 2)) and also because maturation is a continuous process, “an evolution, not an event” (Campion et al., 2013[17]; Picut et al., 2015[83]). In addition, different endpoints may be used by authors for determining sexual maturity (Campion et al., 2013[17]). For more details of puberty and sexual maturity in rats see previous reviews (Ojeda et al., 1980[75]; Rivest, 1991[93]; Blais and Rivest, 2001[14]; Beckman and Feuston, 2003[9]; Marty et al., 2003[62]; Bonthuis et al., 2010[15]; Picut and Remick, 2017[84]; Vidal, 2017[115]; Bell, 2018[10]; Laffan et al., 2018[56]).

#### Adulthood and aging

Rodents > 60 days are considered adult (Hattis et al., 2005[35]). Adulthood in rats is determined according to musculoskeletal maturity (Quinn, 2005[89]), and adult life is after growth and physical development are complete (Roe et al., 1995[94]). However, unlike humans, bone growth never completely stops in rats (Simson and Gold, 1982[102]), and there is no epiphyseal closure in rat's long bones (Kilborn et al., 2002[50]) and therefore tapering of skeletal development is considered as adulthood period (Quinn, 2005[89]), which is 7-8 months in male and female Sprague-Dawley rats (Quinn, 2005[89]). In rodents, peak bone mass is not reached until about 26 weeks of age (Jackson et al., 2017[49]). Rats are aged when their strain has a 50 % survival rate of about 22-24 months and beyond (Hoyer, 1985[43]; Hoyer and Betz, 1988[44]; Stanley and Shetty, 2004[105]; Rao et al., 2005[90]; Simon et al., 2010[101]). Rats between 12 to 21 months are middle-aged (Stanley and Shetty, 2004[105]; Rao et al., 2005[90]), and those between 22-24 until death are aged. As shown in Table 1(Tab. 1), the adulthood period in humans spans from emerging adulthood (18-25 y) to late adulthood (75 years and over) (Bjorklund, 2015[13]). During different periods of adulthood, adult functioning considerably changes in different domains, including physical and mental abilities (Bjorklund, 2015[13]). During the adulthood period, assuming one human year to equal 11.8 rat days (Quinn, 2005[89]), comparable adulthood periods in humans (Bjorklund, 2015[13]) were calculated for rats (Table 1(Tab. 1)). 

The lifespan of laboratory rats has been reported to be 2.5-3.5 years (average 3 years; compared to 80 years in humans) (Quinn, 2005[89]; Sengupta, 2013[99]); it has been reported that longevity is higher in female Wistar rats (2.2-3.7 years) than male ones (1.7-3.2 years) (Schlettwein-Gsell, 1970[97]; Goodrick, 1980[34]). 90 %, 50 % (median lifespan), and 10 % survival age in Wistar rats have been reported to be 1.1-1.4, 1.8-2.1, and 2.5-2.7 years in males and 1.0-1.6,1.9-2.3, and 2.6-2.9 years in females, respectively (Schlettwein-Gsell, 1970[97]). Maximum lifespan means that the last death should be observed in rats; lifespan up to approximately 4.5 years has been reported for male Wistar rats (Lares-Asseff et al., 2006[58]).

### Nutritional behavior

**Figure 1:**
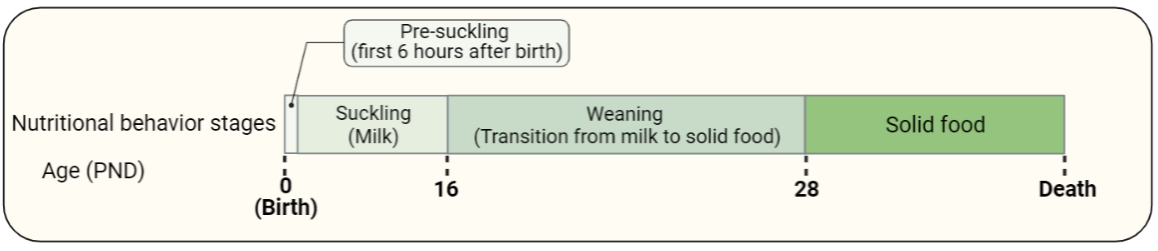
Nutritional behavior in rats includes the pre-suckling period, suckling period, weaning, and solid food consumption.

There are four stages describing the nutritional behavior of rats (Figure 1(Fig. 1)): (1) pre-suckling period, which is the first 6 hours after birth (Mayor and Cuezva, 1985[64]; Ostadalova and Babický, 2012[77]), (2) suckling period, which is exclusively maternal milk intake and takes until PND 16 (Henning et al., 1979[39]; Henning, 1981[38]), (3) weaning, which is a combination of milk and solid food intake (Ostadalova and Babický, 2012[77]) (PND 16-28), and (4) solid food consumption.

In rats, the first 6 hours after birth is called the pre-suckling period (Ostadalova and Babický, 2012[77]). During the pre-suckling period (also called neonatal starvation (Kuma et al., 2004[55])), transplacental nutrient supply is interrupted, and maternal milk nutrition is not yet fully developed, and thus neonates face severe starvation (Kuma et al., 2004[55]; Ostadalova and Babický, 2012[77]). Glucose and lactate derived from the liver and muscle glycogen, are energy sources for pre-suckling newborns. Muscle glycogenolysis provides more contribution during the first 2 hours after birth, and liver glycogenolysis contributes more during 2-6 hours after birth (Mayor and Cuezva, 1985[64]). Thus, during the first 2 hours after birth, lactate can directly be used as an energy source in newborn rats, particularly in the brain, or it may be converted to glucose (Mayor and Cuezva, 1985[64]). Between 3-6-hour after birth, liver gluconeogenesis from lactate increases by 2-fold and contributes to providing glucose as an energy source (Mayor and Cuezva, 1985[64]). In addition, in the pre-suckling period, autophagic degradation of proteins produces amino acids, which may be used as an energy source directly or converted to glucose in the liver (Kuma et al., 2004[55]). This autophagy is transient and reaches its maximum levels 3-6 hours after birth (Kuma et al., 2004[55]; Ostadalova and Babický, 2012[77]). 

After the pre-suckling period, the suckling period (exclusively maternal milk intake) is initiated, and mother's milk is the only food consumed by the rats during the first 16 postnatal days (Henning et al., 1979[39]; Henning, 1981[38]). During the suckling period, neonates consume high fat, low carbohydrate diet from their mothers' milk, and hepatic oxidation of fatty acids provides the bulk of the energy requirements (Henning, 1981[38]; Mayor and Cuezva, 1985[64]). Ketone bodies produced from fatty acid oxidation are energy substrates for the extrahepatic tissues, with the glucose requirements mostly covered by gluconeogenesis (Mayor and Cuezva, 1985[64]). 

In Sprague-Dawley rats, weaning (transition from mother milk to independent ingestion of solid food and water) (Alberts, 2005[3]) begins around PND 14-17 (Redman and Sweney, 1976; Henning et al., 1979[39]; Henning, 1981[38]), gradually increases through PND 23 and completes on PND 26 (Henning et al., 1979[39]) or PND 29 (Redman and Sweney, 1976[91]). Natural weaning occurs between PND 14-30 in Sprague-Dawley rats (with an accelerated phase between PND 18-25) (Redman and Sweney, 1976[91]) and between PND 14-34 in Norway rats (Alberts, 2005[3]). It is common in animal experimentation to wean (separating offspring from the dam) rats at PND 21 (Eckstein et al., 1973[24]; Alberts, 2005[3]; Quinn, 2005[89]; Stoker et al., 2006[107]; McCutcheon and Marinelli, 2009[65]). In the weaning period, a change occurs from a high-fat diet to a high carbohydrate diet concurrent with increased hepatic lipogenesis and increased insulin/glucagon ratio (Mayor and Cuezva, 1985[64]). Transition to solid food is due to insufficient nutritional caloric supply provided by milk for the growth of rats (Ostadalova and Babický, 2012[77]).

## Addressing Rat Developmental Stages in the Literature

Terminology to describe various developmental milestones in rodents is not consistent across publications (Jackson et al., 2017[49]; Picut and Ziejewski, 2018[86]). Male Wistar rats at PND 28 (Kosaka et al., 1987[53]) or 42 (Cunha et al., 2001[20]), male Sprague Dawley rats at PND 36-37 (Ku et al., 2016[54]), male Fisher rats at PND 30 (Swamy and Abraham, 1987[109]) or 45-60 (Delp et al., 1998[23]), and male WKY rats at PND 35 (Silva et al., 2011[100]) have been considered to be juvenil. 9-week old male Wistar rats (Kosaka et al., 1987[53]), 8-week old male Sprague Dawley (Ku et al., 2016[54]), 4-month old male Fisher rats (Swamy and Abraham, 1987[109]), 2-month old female Wistar rats (Pestronk et al., 1980[82]), 6-week old female Sprague Dawley rats (Meyer et al., 2006[66]), and 3-6-month-old Fisher 344 rats (Delp et al., 1998[23]; Stanley and Shetty, 2004[105]; Rao et al., 2005[90]), have been considered young adults. Furthermore, 7-11-month-old Fisher 344 rats (Stanley and Shetty, 2004[105]), 12-month old rats (Hoyer, 1985[43]), 6-month female Sprague Dawley (Meyer et al., 2006[66]), and Wistar rats at ages 21 weeks (~5 months) and beyond (Wang et al., 2004[116]) have been considered as adult rats. Wistar rats at age 24 months (Hoyer, 1985[43]; Hoyer and Betz, 1988[44]) and 18-24 months (Cunha et al., 2001[20]), Sprague Dawley rats at age 12 months (Meyer et al., 2006[66]), and Fisher rats at age 24 months (Delp et al., 1998[23]; Simon et al., 2010[101]), 22 months (Stanley and Shetty, 2004[105]; Rao et al., 2005[90]), and 23-28 months (Swamy and Abraham, 1987[109]) have been considered to be aged.

These data highlight the inconsistencies in reporting the developmental stages of rats. The systematic review in neuroscience research indicated that 42 % of studies defined animals as “adults,” but the papers indicate that the animal's age was not within the adult range (McCutcheon and Marinelli, 2009[65]). A suggestion is that the exact age of the studied animals should be reported (Jackson et al., 2017[49]). According to a survey collected data from researchers that use rodents to model human disease or physiology, rats and mice were primarily used at 8-12 weeks of age and were considered to be adults, and were used as such regardless of the biology being studied (Jackson et al., 2017[49]). The definition of an adult was mainly related to the sexual maturity of rodents, not the development of the system under examination (Jackson et al., 2017[49]). This range (8-12 weeks) encompasses ongoing development in some systems, e.g., brain development (McCutcheon and Marinelli, 2009[65]), which affects the outcome of experiments and can lead to misinterpretation of the data obtained (Jackson et al., 2017[49]). For example, most humans with sepsis are over the age of 50, whereas most mice used in sepsis research are < 3 months old, this mismatch causes misinterpretation of the data obtained as the immune response to infection is age-dependent (Fink, 2014[27]; Starr and Saito, 2014[106]). In addition, even though many neurological disorders affect the elderly, most studies have used young adult animals, with only 2.2-10.5 % of 10,3269 rodents used in neurological disorder studies included aged rodents (Sun et al., 2020[108]). A systematic review of animal experiments in neuroscience research indicates that 75 % of studies used young animals, 20 % used adult animals, and 5 % did not specify animal age, indicating bias towards using young animals (McCutcheon and Marinelli, 2009[65]).

## Body Weight Changes in Rats

The Wistar and Sprague Dawley rats' birth weight ranges from 5-7 g (Gille et al., 1994-1996[32]; Tinwell et al., 2002[112]; Alberts, 2005[3]; Sengupta, 2013[99]; Santiago et al., 2015[96]). Body weight growth in rats has two stages: (1) development to maturity in which growth rate is high, and all parts of the body grow, (2) post-maturity growth in which the growth rate is lower than the previous stage (Pahl, 1969[78]). Growth duration in male and female Wistar rats has been reported to be 13.5±0.4 months and 19.3±0.5 months, respectively (Goodrick, 1980[34]).

### Pre-maturity growth

During the first two months of postnatal life, rats' body weight changes considerably (McCutcheon and Marinelli, 2009[65]), reaching 20 g by PND 10 and 30 g by PND 15 (Alberts, 2005[3]). In rats, body growth is not linear during early postnatal development (Ostadalova and Babický, 2012[77]). The growth pattern is similar in male and female Wistar rats up to PND 21, and the body weights of male and female Wistar rats are similar before PND 26 (Pullen, 1976[88]). Similar results have been reported in Sprague Dawley rats; body weights of male Sprague Dawley rats in PND 0, 7, 21 are about 5.6, 12.5, and 38.3 g, and in female Sprague Dawley rats are about 5.4, 12.4, and 37.9 g, respectively (Somm et al., 2012[103]). In rats, from PND 30 onwards, the differences between male and female body weights steadily increase (Pahl, 1969[78]). In support, it has been reported that body weights in male and female Sprague Dawley rats are about 40-49 g in PND 21 and 73-84 g in PND 28 that increases to 200 and 322 g in male rats and 167 and 219 g in female rats in PND 42 and 56, respectively (Picut et al., 2014[85]; Turnbull et al., 2021[114]). Maximum growth rates occur at PND 34-38 in female (3.5 g/day) and PND 42-45 in male (5.9 g/day) rats (Pahl, 1969[78]; Gille et al., 1994-1996[32]; Watson et al., 2006[117]). 

### Post-maturity growth

In male Wistar rats, initial rapid growth is observed before PND 60, and after that, body weight gain occurs at a slower rate (Novelli et al., 2007[71]). Maximum body weight in male and female Wistar rats has been reported to be 677.3±9.2 g and 463.3±8.6 g, respectively (Goodrick, 1980[34]) and is attained by PND 100 in males and slightly sooner in females (Pullen, 1976[88]). In Alderley Park (AP) rats, which are Wistar-derived, about 75 % of growth occurs until the PND 154 to PND 168 and the remaining 25 % until PND 560 (Tucker, 1997[113]). Weight gain is negligible after PND 150-170 in rats (Gille et al., 1994-1996[32]), and no differences are observed between the rate of body weight gain at PND 120 and PND 150 (Novelli et al., 2007[71]). The body weight of Wistar rats increased steadily from PND 147 to PND 553 in male rats and from PND 147 to PND 735 in female rats (Wang et al., 2004[116]). It has also been reported that in male Wistar rats, body weight increases up to PND 483 and has a slower rate of rising between PND 483 to PND 938, and after that, body weight starts to decrease (Nistiar et al., 2012[70]). In Sprague Dawley rats, initial rapid growth is observed until PND 168, after which growth continues slower until 18-24 months that reach peaks (about 365 g in female rats and 597 g in male rats). Beyond 24 months, the rats maintain their weight or reveal a modest decrease (Altun et al., 2007[5]).

## Body Weight: How to Report

There is no standard guideline for reporting animal body weights in the literature, and the time interval for data analysis depends on the researcher's decision. It has been proposed that rats have a growth phase from birth until the end of the 14^th^ week and, after that, have a maintenance phase in which the growth rate is lower (Hoffman et al., 2002[40]). It has been suggested that during the growth phase, body weights should be measured every week and during the maintenance phase every two weeks (Hoffman et al., 2002[40]). It would be better to statistically analyze and report the data on body weights weekly for the first four weeks of the animal's age (n=4), then every two weeks at weeks 5, 7, 9, 11, and 13 from a three-week moving average (n=5); for example, the three-week moving average at week 5 is the average of week 5, week 4 (one week before), and week 6 (one week after). During the maintenance phase, every four weeks at weeks 16, 20, and 24 from a 5-week moving average (n=3), followed by every 14 weeks at weeks 33, 47, 61, 75, 89 from a 15-week moving average (n=5) and the last point is the midpoint from week 96 to the end of the study (total n=18) (Hoffman et al., 2002[40]). For statistical analysis, one repeated measure ANOVA is used for each growth phase; this approach decreases the number of comparisons and, therefore chance of making false-positive claims (Hoffman et al., 2002[40]). However, using the moving average as a smoothing data technique has been criticized as it can produce spurious signals that look real (http://wmbriggs.com/post/195/) and are not suitable for statistical analyses (https://www.graphpad.com/guides/prism/latest/curve-fitting/reg_dont_fit_a_model_to_smoothed_d.htm).

Another problem with reporting data on animal body weight is that in most studies, particularly when there is repeated measure data, body weights are reported in the article's Results section as a graph. Although it seems the right and appropriate way of reporting the data, it precludes it from entering secondary analysis such as meta-analyses that are informative tools for translating basic sciences into clinical practice (Bahadoran et al., 2020[7]). There are simple ways, such as using Adobe Photoshop software for extracting data from graphs (Gheibi et al., 2019[31]), and an alternative suggestion is to provide such data in a Supplementary Table. It has also been suggested that if body weight is not an intended outcome of the study, at least the animals' initial and final body weights are reported. Also, in long-term studies, the dose of a drug used in the drinking water needs to be readjusted according to the animal's body weight during the study period. 

## Body Weight Measurement: What Does It Tell Us?

Besides being the primary outcome in some studies (Wang et al., 2004[116]), animal body weights are measured during *in vivo* animal studies to assess the animals' overall health (Hoffman et al., 2008[41]). Body weights provide an objective measure of laboratory animals' health and/or development (Hawkins, 2002[37]). Failure to maintain body weight in adults or failure to reach expected body weight in growing animals indicates abnormalities (Morton and Griffiths, 1985[67]). The body weight of laboratory animals is an indicator of animal distress (Talbot et al., 2020[110]) and is used as an objective sign of pain and discomfort (Morton and Griffiths, 1985[68]; Baumans et al., 1994[8]). In animal studies, body weight loss >20 % is considered severe suffering and is a potential parameter for human endpoint decisions unless a severe outcome is predicted (Morton, 2000[67]). In addition, weight loss of up to 20 % along with food and water consumption < 40 % of normal for 72 h has been considered as a moderate sign of pain and discomfort in laboratory animals and weight loss > 25 % along with food and water consumption < 40 % of normal for seven days or anorexia is a substantial sign of pain and discomfort in laboratory animals (Baumans et al., 1994[8]).

In addition, in animal experimentations, some outcomes including body mass index (BMI), (Novelli et al., 2007[71]) Lee index (Lee, 1929[59]), adiposity index (Li et al., 1997[61]), specific rate of body mass gain (Novelli et al., 2007[71]), feed efficiency ratio (Hsu and Yen, 2007[45]; Yeon et al., 2015[118]), and efficiency of food utilization (EFU) for BW (EFU_BW_) (Bernardis et al., 1982[11]), efficiency of food utilization for Lee index (EFU_Lee_) (Bernardis et al., 1982[11]), and estimated glomerular filtration rats (eGFR) (Besseling et al., 2021[12]) are calculated using body weight as a variable (Table 3(Tab. 3); References in Table 3: Bernardis et al., 1982[11]; Besseling et al., 2021[12]; Hsu and Yen, 2007[45]; Lee, 1929[59]; Li et al., 1997[61]; Novelli et al., 2007[71]; Yeon et al., 2015[118]).

## Conclusion

Animal age (McCutcheon and Marinelli, 2009[65]) and body weight (Alfaro, 2005[4]) should be reported in scientific papers that use animals for experimentations. In addition, the reasoning for age choice and rodent age relevant to the human disease being studied needs to be provided in reporting animal studies. Such ancillary variables help improve the analysis and interpretation of the data, may lead to new hypothesis generation, offer a possible explanation for outliers, and prevent using more than the minimum number of animals needed (Gaines Das, 2002[29]). Furthermore, better reporting of animal studies increases the integrity of animal research, enhancing the chance of extrapolating from animals to humans. Finally, rats are not humans (Cunningham, 2002[21]) or miniature humans (Andreollo et al., 2012[6]), which should be considered when translating animal data to humans.

## Notes

Sajad Jeddi and Khosrow Kashfi (Department of Molecular, Cellular and Biomedical Sciences, Sophie Davis School of Biomedical Education, City University of New York School of Medicine, New York, NY 10031 USA; Tel: +1 212-650-6641, E-mail: Kashfi@med.cuny.edu) contributed equally as corresponding author.

## Conflict of interest

The authors declare that they have no competing interests.

## Acknowledgements

This study was supported by Shahid Beheshti University of Medical Sciences [grant No. 29431-1], Tehran, Iran. In addition, KK: Supported in part by the National Institutes of Health [R24 DA018055; R01GM123508] and the Professional Staff Congress-City University of New York (PSC-CUNY) [TRADB-49-271]. 

## Figures and Tables

**Table 1:**
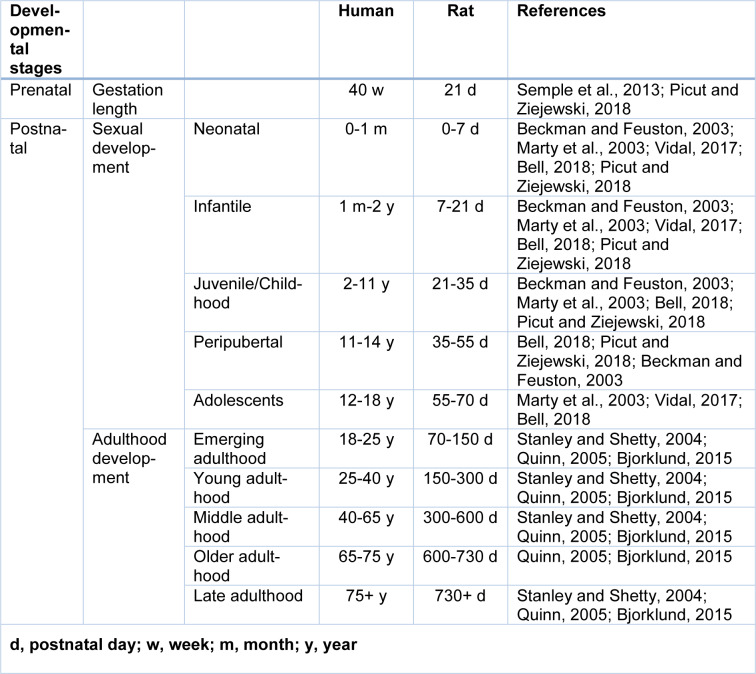
Comparable ages of the developmental stages in humans and rats

**Table 2:**
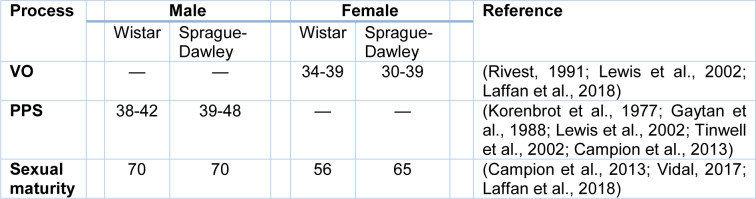
Age (postnatal day) at the vaginal opening (VO) and preputial separation (PPS) as indicators of the onset of puberty in male and female Wistar and Sprague-Dawley rats

**Table 3:**
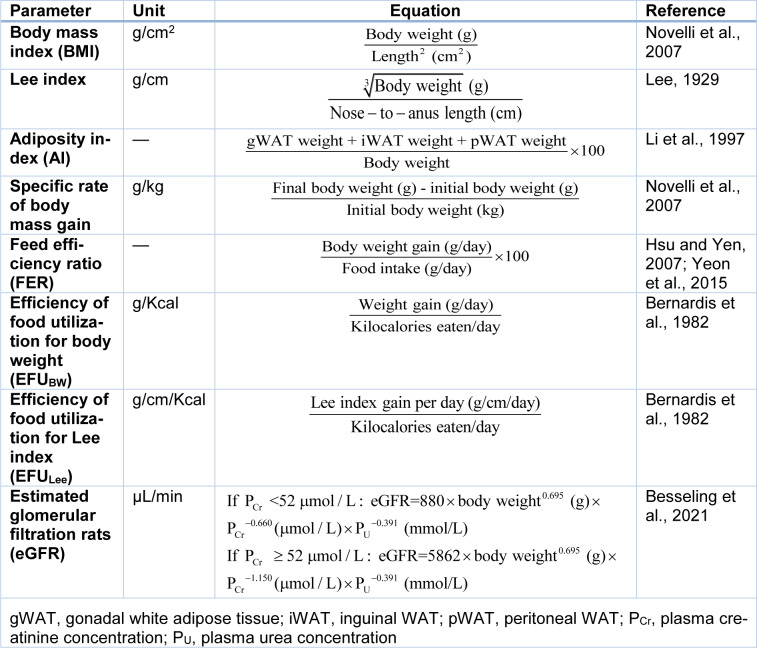
Some anthropometric, nutritional, and functional variables calculated in rats using body weight as a variable

## References

[1] Agoston DV (2017). How to translate time? The temporal aspect of human and rodent biology. Front Neurol.

[2] Akhtar A (2015). The flaws and human harms of animal experimentation. Camb Q Healthc Ethics.

[3] Alberts JY, Whishaw IQ, Kolb B (2005). Infancy. The behavior of the laboratory rat: a handbook with tests.

[4] Alfaro V (2005). Specification of laboratory animal use in scientific articles: current low detail in the journals' instructions for authors and some proposals. Methods Find Exp Clin Pharmacol.

[5] Altun M, Bergman E, Edström E, Johnson H, Ulfhake B (2007). Behavioral impairments of the aging rat. Physiol Behav.

[6] Andreollo NA, Santos EF, Araújo MR, Lopes LR (2012). Rat's age versus human's age: what is the relationship?. Arq Bras Cir Dig.

[7] Bahadoran Z, Mirmiran P, Kashfi K, Ghasemi A (2020). Importance of systematic reviews and meta-analyses of animal studies: challenges for animal-to-human translation. J Am Assoc Lab Anim Sci.

[8] Baumans V, Brain PF, Brugere H, Clausing P, Jeneskog T, Perretta G (1994). Pain and distress in laboratory rodents and lagomorphs. Report of the Federation of European Laboratory Animal Science Associations (FELASA) Working Group on Pain and Distress accepted by the FELASA Board of Management November 1992. Lab Anim.

[9] Beckman DA, Feuston M (2003). Landmarks in the development of the female reproductive system. Birth Defects Res B Dev Reprod Toxicol.

[10] Bell MR (2018). Comparing postnatal development of gonadal hormones and associated social behaviors in rats, mice, and humans. Endocrinology.

[11] Bernardis LL, Luboshitsky R, Bellinger LL, McEwen G (1982). Nutritional studies in the weanling rat with normophagic hypothalamic obesity. J Nutr.

[12] Besseling PJ, Pieters TT, Nguyen ITN, de Bree PM, Willekes N, Dijk AH (2021). A plasma creatinine- and urea-based equation to estimate glomerular filtration rate in rats. Am J Physiol Renal Physiol.

[13] Bjorklund BR (2015). The journey of adulthood.

[14] Blais V, Rivest S (2001). Inhibitory action of nitric oxide on circulating tumor necrosis factor-induced NF-kappaB activity and COX-2 transcription in the endothelium of the brain capillaries. J Neuropathol Exp Neurol.

[15] Bonthuis PJ, Cox KH, Searcy BT, Kumar P, Tobet S, Rissman EF (2010). Of mice and rats: key species variations in the sexual differentiation of brain and behavior. Front Neuroendocrinol.

[16] Buwalda B, Geerdink M, Vidal J, Koolhaas JM (2011). Social behavior and social stress in adolescence: a focus on animal models. Neurosci Biobehav Rev.

[17] Campion SN, Carvallo FR, Chapin RE, Nowland WS, Beauchamp D, Jamon R (2013). Comparative assessment of the timing of sexual maturation in male Wistar Han and Sprague-Dawley rats. Reprod Toxicol.

[18] Clause BT (1993). The Wistar Rat as a right choice: establishing mammalian standards and the ideal of a standardized mammal. J Hist Biol.

[19] Collins FS, Tabak LA (2014). Policy: NIH plans to enhance reproducibility. Nature.

[20] Cunha RA, Almeida T, Ribeiro JA (2001). Parallel modification of adenosine extracellular metabolism and modulatory action in the hippocampus of aged rats. J Neurochem.

[21] Cunningham ML (2002). A mouse is not a rat is not a human: species differences exist. Toxicol Sci.

[22] De Silva NK, Tschirhart J (2016). Puberty-defining normal and understanding abnormal. Curr Treat Opt Pediatr.

[23] Delp MD, Evans MV, Duan C (1998). Effects of aging on cardiac output, regional blood flow, and body composition in Fischer-344 rats. J Appl Physiol (1985).

[24] Eckstein B, Golan R, Shani J (1973). Onset of puberty in the immature female rat induced by 5 -androstane-3 ,17 -diol. Endocrinology.

[25] Ericsson AC, Crim MJ, Franklin CL (2013). A brief history of animal modeling. Missouri Med.

[26] Finegood DT, Scaglia L, Bonner-Weir S (1995). Dynamics of beta-cell mass in the growing rat pancreas. Estimation with a simple mathematical model. Diabetes.

[27] Fink MP (2014). Animal models of sepsis. Virulence.

[28] Flórez-Vargas O, Brass A, Karystianis G, Bramhall M (2016). Bias in the reporting of sex and age in biomedical research on mouse models. Elife.

[29] Gaines Das RE (2002). Role of ancillary variables in the design, analysis, and interpretation of animal experiments. ILAR J.

[30] Gaytan F, Bellido C, Aguilar R, Aguilar E (1988). Balano-preputial separation as an external sign of puberty in the rat: correlation with histologic testicular data. Andrologia.

[31] Gheibi S, Mahmoodzadeh A, Kashfi K (2019). Data extraction from graphs using adobe photoshop: applications for meta-analyses. Int J Endocrinol Metab.

[32] Gille U, Salomon F-V, Rieck O, Gericke A, Ludwig B (1994-1996). Growth in rats (Rattus norvegicus Berkenhout). 1. Growth of body mass: A comparison of different models. J Exp Anim Sci (Germany).

[33] Goodman J, Chandna A, Roe K (2015). Trends in animal use at US research facilities. J Med Ethics.

[34] Goodrick CL (1980). Effects of long-term voluntary wheel exercise on male and female Wistar rats. I. Longevity, body weight, and metabolic rate. Gerontology.

[35] Hattis D, Goble R, Chu M (2005). Age-related differences in susceptibility to carcinogenesis. II. Approaches for application and uncertainty analyses for individual genetically acting carcinogens. Environ Health Perspect.

[36] Hatton GB, Yadav V, Basit AW, Merchant HA (2015). Animal Farm: considerations in animal gastrointestinal physiology and relevance to drug delivery in humans. J Pharm Sci.

[37] Hawkins P (2002). Recognizing and assessing pain, suffering and distress in laboratory animals: a survey of current practice in the UK with recommendations. Lab Anim.

[38] Henning SJ (1981). Postnatal development: coordination of feeding, digestion, and metabolism. Am J Physiol.

[39] Henning SJ, Chang SS, Gisel EG (1979). Ontogeny of feeding controls in suckling and weanling rats. Am J Physiol.

[40] Hoffman WP, Ness DK, van Lier RB (2002). Analysis of rodent growth data in toxicology studies. Toxicol Sci.

[41] Hoffman WP, Recknor J, Lee C (2008). Overall type I error rate and power of multiple Dunnett's tests on rodent body weights in toxicology studies. J Biopharm Stat.

[42] Hooijmans CR, Leenaars M, Ritskes-Hoitinga M (2010). A gold standard publication checklist to improve the quality of animal studies, to fully integrate the Three Rs, and to make systematic reviews more feasible. Alternatives to laboratory animals: ATLA.

[43] Hoyer S (1985). The effect of age on glucose and energy metabolism in brain cortex of rats. Arch Gerontol Geriat.

[44] Hoyer S, Betz K (1988). Abnormalities in glucose and energy metabolism are more severe in the hippocampus than in cerebral cortex in postischemic recovery in aged rats. Neurosci Lett.

[45] Hsu CL, Yen GC (2007). Effect of gallic acid on high fat diet-induced dyslipidaemia, hepatosteatosis and oxidative stress in rats. Br J Nutr.

[46] Hudson-Shore M (2016). Statistics of scientific procedures on living animals Great Britain 2015 - highlighting an ongoing upward trend in animal use and missed opportunities. ATLA.

[47] Ihedioha JI, Noel-Uneke OA, Ihedioha TE (2013). Reference values for the serum lipid profile of albino rats (Rattus norvegicus) of varied ages and sexes. Comp Clin Pathol.

[48] Ioannidis JP (2012). Extrapolating from animals to humans. Sci Transl Med.

[49] Jackson SJ, Andrews N, Ball D, Bellantuono I, Gray J, Hachoumi L (2017). Does age matter? The impact of rodent age on study outcomes. Lab Anim.

[50] Kilborn SH, Trudel G, Uhthoff H (2002). Review of growth plate closure compared with age at sexual maturity and lifespan in laboratory animals. Contemp Top Lab Anim Sci.

[51] Kilkenny C, Parsons N, Kadyszewski E, Festing MF, Cuthill IC, Fry D (2009). Survey of the quality of experimental design, statistical analysis and reporting of research using animals. PloS One.

[52] Korenbrot CC, Huhtaniemi IT, Weiner RI (1977). Preputial separation as an external sign of pubertal development in the male rat. Biol Reprod.

[53] Kosaka T, Kosaka K, Hama K, Wu JY, Nagatsu I (1987). Differential effect of functional olfactory deprivation on the GABAergic and catecholaminergic traits in the rat main olfactory bulb. Brain Res.

[54] Ku KM, Weir RK, Silverman JL, Berman RF, Bauman MD (2016). Behavioral phenotyping of juvenile long-evans and sprague-dawley rats: implications for preclinical models of autism spectrum disorders. PloS One.

[55] Kuma A, Hatano M, Matsui M, Yamamoto A, Nakaya H, Yoshimori T (2004). The role of autophagy during the early neonatal starvation period. Nature.

[56] Laffan SB, Posobiec LM, Uhl JE, Vidal JD (2018). Species comparison of postnatal development of the female reproductive system. Birth Defects Res.

[57] Lane PH (1997). Age of onset of streptozocin diabetes determines the renal structural response in the rat. Pediatr Res.

[58] Lares-Asseff I, Camacho GA, Guillé AJ, Toledo AR, Trujillo F, Reyes RE (2006). Changes in acetylator phenotype over the lifespan in the Wistar rat. Mech Ageing Dev.

[59] Lee MO (1929). Determination of the surface area of the white rat with its application to the expression of metabolic results. Am J Physiol Legacy Content.

[60] Lewis EM, Barnett JF, Freshwater L, Hoberman AM, Christian MS (2002). Sexual maturation data for Crl Sprague-Dawley rats: criteria and confounding factors. Drug Chem Toxicol.

[61] Li H, Matheny M, Nicolson M, Tumer N, Scarpace PJ (1997). Leptin gene expression increases with age independent of increasing adiposity in rats. Diabetes.

[62] Marty MS, Chapin RE, Parks LG, Thorsrud BA (2003). Development and maturation of the male reproductive system. Birth Defects Res B Dev Reprod Toxicol.

[63] Masiello P, De Paoli AA, Bergamini E (1979). Influence of age on the sensitivity of the rat to streptozotocin. Hormone Res.

[64] Mayor F, Cuezva JM (1985). Hormonal and metabolic changes in the perinatal period. Biol Neonate.

[65] McCutcheon JE, Marinelli M (2009). Age matters. Eur J Neurosci.

[66] Meyer RA, Desai BR, Heiner DE, Fiechtl J, Porter S, Meyer MH (2006). Young, adult, and old rats have similar changes in mRNA expression of many skeletal genes after fracture despite delayed healing with age. J Orthop Res.

[67] Morton DB (2000). A systematic approach for establishing humane endpoints. ILAR J.

[68] Morton DB, Griffiths PH (1985). Guidelines on the recognition of pain, distress and discomfort in experimental animals and an hypothesis for assessment. Veter Rec.

[69] Nagendrababu V, Kishen A, Murray P, Nekoofar MH, de Figueiredo JAP, Priya E (2021). PRIASE 2021 guidelines for reporting animal studies in Endodontology: explanation and elaboration. Int Endodont J.

[70] Nistiar F, Racz O, Lukacinova A, Hubkova B, Novakova J, Lovasova E (2012). Age dependency on some physiological and biochemical parameters of male Wistar rats in controlled environment. J Environ Sci Health A.

[71] Novelli EL, Diniz YS, Galhardi CM, Ebaid GM, Rodrigues HG, Mani F (2007). Anthropometrical parameters and markers of obesity in rats. Lab Anim.

[72] NRC, National Research Council (2011). Guidance for the description of animal research in scientific publications. https://www.nap.edu/catalog/13241/guidance-for-the-description-of-animal-research-in-scientific-publications.

[73] Nuñez JL, Alt JJ, McCarthy MM (2003). A new model for prenatal brain damage. I. GABAA receptor activation induces cell death in developing rat hippocampus. Exp Neurol.

[74] O'Connor AM, Sargeant JM (2014). Critical appraisal of studies using laboratory animal models. ILAR J.

[75] Ojeda SR, Andrews WW, Advis JP, White SS (1980). Recent advances in the endocrinology of puberty. Endocr Rev.

[76] Osborne N, Avey MT, Anestidou L, Ritskes-Hoitinga M (2018). Improving animal research reporting standards: HARRP, the first step of a unified approach by ICLAS to improve animal research reporting standards worldwide. EMBO Rep.

[77] Ostadalova I, Babický A (2012). Periodization of the early postnatal development in the rat with particular attention to the weaning period. Physiol Res.

[78] Pahl PJ (1969). Growth curves for body weight of the laboratory rat. Aust J Biol Sci.

[79] Pasquali P (2018). The importance of animal models in research. Res Veter Sci.

[80] Percie du Sert N, Hurst V, Ahluwalia A (2020). The ARRIVE guidelines 2.0: updated guidelines for reporting animal research. PLoS Biol.

[81] Perrin S (2014). Preclinical research: Make mouse studies work. Nature.

[82] Pestronk A, Drachman DB, Griffin JW (1980). Effects of aging on nerve sprouting and regeneration. Exp Neurol.

[83] Picut CA, Dixon D, Simons ML, Stump DG, Parker GA, Remick AK (2015). Postnatal ovary development in the rat: morphologic study and correlation of morphology to neuroendocrine parameters. Toxicol Pathol.

[84] Picut CA, Remick AK (2017). Impact of age on the male reproductive system from the pathologist's perspective. Toxicol Pathol.

[85] Picut CA, Remick AK, Asakawa MG, Simons ML, Parker GA (2014). Histologic features of prepubertal and pubertal reproductive development in female Sprague-Dawley rats. Toxicol Pathol.

[86] Picut CA, Ziejewski MK (2018). Comparative aspects of pre- and postnatal development of the male reproductive system. Birth Defects Res.

[87] Pound P, Ebrahim S, Sandercock P, Bracken MB, Roberts I (2004). Where is the evidence that animal research benefits humans?. BMJ (Clin Res ed).

[88] Pullen AH (1976). A parametric analysis of the growing CFHB (Wistar) rat. J Anat.

[89] Quinn R (2005). Comparing rat's to human's age: how old is my rat in people years?. Nutrition (Burbank, Los Angeles County, Calif).

[90] Rao MS, Hattiangady B, Abdel-Rahman A, Stanley DP, Shetty AK (2005). Newly born cells in the ageing dentate gyrus display normal migration, survival and neuronal fate choice but endure retarded early maturation. Eur J Neurosci.

[91] Redman RS, Sweney LR (1976). Changes in diet and patterns of feeding activity of developing rats. J Nutr.

[92] Reed DR, Duke FF, Ellis HK, Rosazza MR, Lawler MP, Alarcon LK (2011). Body fat distribution and organ weights of 14 common strains and a 22-strain consomic panel of rats. Physiol Behav.

[93] Rivest RW (1991). Sexual maturation in female rats: hereditary, developmental and environmental aspects. Experientia.

[94] Roe FJ, Lee PN, Conybeare G, Kelly D, Matter B, Prentice D (1995). The Biosure Study: influence of composition of diet and food consumption on longevity, degenerative diseases and neoplasia in Wistar rats studied for up to 30 months post weaning. Food Chem Toxicol.

[95] Romijn HJ, Hofman MA, Gramsbergen A (1991). At what age is the developing cerebral cortex of the rat comparable to that of the full-term newborn human baby?. Early Hum Dev.

[96] Santiago HA, De Pierro LR, Reis RM, Caluz AG, Ribeiro VB, Volpon JB (2015). Allometric relationships among body mass, MUZZLE-tail length, and tibia length during the growth of Wistar rats. Acta Cir Bras.

[97] Schlettwein-Gsell D (1970). Survival curves of an old age rat colony. Gerontologia.

[98] Semple BD, Blomgren K, Gimlin K, Ferriero DM, Noble-Haeusslein LJ (2013). Brain development in rodents and humans: Identifying benchmarks of maturation and vulnerability to injury across species. Progr Neurobiol.

[99] Sengupta P (2013). The laboratory rat: relating its age with human's. Int J Prev Med.

[100] Silva E, Serrão MP, Soares-da-Silva P (2011). Age-dependent effect of ouabain on renal Na+,K+-ATPase. Life Sci.

[101] Simon NW, LaSarge CL, Montgomery KS, Williams MT, Mendez IA, Setlow B (2010). Good things come to those who wait: attenuated discounting of delayed rewards in aged Fischer 344 rats. Neurobiol Aging.

[102] Simson EL, Gold RM (1982). The Lee Obesity Index vindicated?. Physiol Behav.

[103] Somm E, Vauthay DM, Guérardel A, Toulotte A, Cettour-Rose P, Klee P (2012). Early metabolic defects in dexamethasone-exposed and undernourished intrauterine growth restricted rats. PloS One.

[104] Squeglia LM, Boissoneault J, Van Skike CE, Nixon SJ, Matthews DB (2014). Age-related effects of alcohol from adolescent, adult, and aged populations using human and animal models. Alcoholism, clinical and experimental research.

[105] Stanley DP, Shetty AK (2004). Aging in the rat hippocampus is associated with widespread reductions in the number of glutamate decarboxylase-67 positive interneurons but not interneuron degeneration. J Neurochem.

[106] Starr ME, Saito H (2014). Sepsis in old age: review of human and animal studies. Aging Dis.

[107] Stoker TE, Ferrell JM, Laws SC, Cooper RL, Buckalew A (2006). Evaluation of ammonium perchlorate in the endocrine disruptor screening and testing program's male pubertal protocol: ability to detect effects on thyroid endpoints. Toxicology.

[108] Sun M, McDonald SJ, Brady RD, Collins-Praino L, Yamakawa GR, Monif M (2020). The need to incorporate aged animals into the preclinical modeling of neurological conditions. Neurosci Biobehav Rev.

[109] Swamy MS, Abraham EC (1987). Lens protein composition, glycation and high molecular weight aggregation in aging rats. Invest Ophthal Vis Sci.

[110] Talbot SR, Biernot S, Bleich A (2020). Defining body-weight reduction as a humane endpoint: a critical appraisal. Lab Anim.

[111] Taylor K, Gordon N, Langley G, Higgins W (2008). Estimates for worldwide laboratory animal use in 2005. ATLA.

[112] Tinwell H, Haseman J, Lefevre PA, Wallis N, Ashby J (2002). Normal sexual development of two strains of rat exposed in utero to low doses of bisphenol A. Toxicol Sci.

[113] Tucker MJ (1997). Diseases of the wistar rat.

[114] Turnbull D, Jack MM, Coder PS, Picut CA, Rodricks JV (2021). Extended One-Generation Reproductive Toxicity (EOGRT) study of benzoic acid in Sprague Dawley rats. Regul Toxicol Pharmacol.

[115] Vidal JD (2017). The impact of age on the female reproductive system. Toxicol Pathol.

[116] Wang C, Weindruch R, Fernández JR, Coffey CS, Patel P, Allison DB (2004). Caloric restriction and body weight independently affect longevity in Wistar rats. Int J Obes Relat Metab Disord.

[117] Watson RE, Desesso JM, Hurtt ME, Cappon GD (2006). Postnatal growth and morphological development of the brain: a species comparison. Birth Defects Res B Dev Reprod Toxicol.

[118] Yeon SJ, Hong GE, Kim CK, Park WJ, Kim SK, Lee CH (2015). Effects of yogurt containing fermented pepper juice on the body fat and cholesterol level in high fat and high cholesterol diet fed rat. Korean J Food Sci Anim Res.

[119] Yousefzadeh N, Kashfi K, Jeddi S, Ghasemi A (2020). Ovariectomized rat model of osteoporosis: a practical guide. EXCLI J.

